# Innate Immune Responses to Acute Viral Infection During Pregnancy

**DOI:** 10.3389/fimmu.2020.572567

**Published:** 2020-09-30

**Authors:** Emily F. Cornish, Iva Filipovic, Fredrika Åsenius, David J. Williams, Thomas McDonnell

**Affiliations:** ^1^Elizabeth Garrett Anderson Institute for Women's Health, University College London, London, United Kingdom; ^2^Center for Infectious Medicine, Department of Medicine Huddinge, Karolinska Institute, Stockholm, Sweden; ^3^Department of Biochemical Engineering, University College London, London, United Kingdom

**Keywords:** pregnancy, innate antiviral immunity, Lassa virus, Ebola virus, dengue virus, hepatitis E, influenza virus, emerging coronavirus

## Abstract

Immunological adaptations in pregnancy allow maternal tolerance of the semi-allogeneic fetus but also increase maternal susceptibility to infection. At implantation, the endometrial stroma, glands, arteries and immune cells undergo anatomical and functional transformation to create the decidua, the specialized secretory endometrium of pregnancy. The maternal decidua and the invading fetal trophoblast constitute a dynamic junction that facilitates a complex immunological dialogue between the two. The decidual and peripheral immune systems together assume a pivotal role in regulating the critical balance between tolerance and defense against infection. Throughout pregnancy, this equilibrium is repeatedly subjected to microbial challenge. Acute viral infection in pregnancy is associated with a wide spectrum of adverse consequences for both mother and fetus. Vertical transmission from mother to fetus can cause developmental anomalies, growth restriction, preterm birth and stillbirth, while the mother is predisposed to heightened morbidity and maternal death. A rapid, effective response to invasive pathogens is therefore essential in order to avoid overwhelming maternal infection and consequent fetal compromise. This sentinel response is mediated by the innate immune system: a heritable, highly evolutionarily conserved system comprising physical barriers, antimicrobial peptides (AMP) and a variety of immune cells—principally neutrophils, macrophages, dendritic cells, and natural killer cells—which express pattern-receptors that detect invariant molecular signatures unique to pathogenic micro-organisms. Recognition of these signatures during acute infection triggers signaling cascades that enhance antimicrobial properties such as phagocytosis, secretion of pro-inflammatory cytokines and activation of the complement system. As well as coordinating the initial immune response, macrophages and dendritic cells present microbial antigens to lymphocytes, initiating and influencing the development of specific, long-lasting adaptive immunity. Despite extensive progress in unraveling the immunological adaptations of pregnancy, pregnant women remain particularly susceptible to certain acute viral infections and continue to experience mortality rates equivalent to those observed in pandemics several decades ago. Here, we focus specifically on the pregnancy-induced vulnerabilities in innate immunity that contribute to the disproportionately high maternal mortality observed in the following acute viral infections: Lassa fever, Ebola virus disease (EVD), dengue fever, hepatitis E, influenza, and novel coronavirus infections.

## Introduction

Pregnancy creates a unique immunological paradox: the maternal immune system must undergo complex adaptations to permit tolerance of the semi-allogeneic fetus while simultaneously maintaining robust defenses against invasive pathogens. Initial theories of maternal-fetal tolerance proposed that a temporary state of maternal immunosuppression was vital to allow successful implantation and development of a pregnancy (1–3). With the advent of technologies including microscopy, advanced cytometry and single-cell sequencing, these models have been superseded by new data that suggest a tightly regulated balance between inflammatory and tolerogenic states during the “immune chronology” of normal pregnancy (4–8). Longitudinal studies of peripheral, decidual, and amniotic fluid cytokine profiles and immune cell subsets demonstrate that a pro-inflammatory environment predominates during early trophoblast invasion and at parturition, while the second and third trimesters require an anti-inflammatory bias to facilitate fetal growth (5, 8–11). The balance between innate and adaptive immunity shifts in favor of innate mechanisms, particularly in the first trimester; as pregnancy progresses, silencing of chemokine genes inhibits accumulation of effector T cells in the decidua, peripheral B cells are depleted and pregnancy-specific hormones skew B cell polarization toward a tolerogenic IL-10-producing phenotype (12–14).

These changes are orchestrated by the sentinel innate immune cells of the maternal decidua—neutrophils, macrophages, dendritic cells, and natural killer cells—and their molecular interactions with invading fetal trophoblast at the maternal-fetal interface. Activation of decidual innate immunity is crucial in the establishment of a pregnancy-specific immune environment: it recruits additional populations of leukocytes to the decidua, educates adaptive cells to refine appropriate effector and memory responses, and modulates the phenotype and functions of peripheral immune cells (15–18). Dysregulation of this complex bi-directional relationship has been implicated in several obstetric and perinatal complications, including recurrent miscarriage, pre-eclampsia, fetal growth restriction, chorioamnionitis, and preterm birth (19–26).

The corollary of this capacity for immunological tolerance is an increased susceptibility to infection (27–29). The innate cells that mediate maternal-fetal crosstalk and induction of tolerance also constitute the frontier of defense against infection through a wide repertoire of effector mechanisms (30–32). Chief amongst these are the expression of pattern-recognition receptors that detect pathogen-specific molecular signatures and soluble mediators such as the complement system. The World Health Organization estimates that sepsis accounts for 10.7% of maternal deaths globally (33) and there is evidence of a particular vulnerability to acute viral infection. For example, pregnant women suffered disproportionately high mortality rates in the influenza pandemics of 1918, 1957, and 2009 (34–36), and hepatitis E, typically a mild and self-limiting illness, has a 26% case-fatality rate in pregnant women (28). These disparities are likely to arise from a combination of immunological, hormonal, and physiological adaptations that are specific to pregnancy (37–39).

Viral infection in pregnancy carries four distinct risks:

1. Adverse pregnancy outcomes: acute viral infection is consistently associated with a broad spectrum of obstetric complications [reviewed in (40–42)]
2. Acute severe maternal disease with consequent morbidity and/or mortality
3. Vertical transmission to the fetus (during pregnancy), resulting in congenital infection that can cause intrauterine death or permanent disability
4. Perinatal transmission to the fetus (during delivery), which can cause severe neonatal disease.

Although congenital infection is a major public health concern, this article will not cover viruses that cause fetal damage through vertical or horizontal transmission as these have been recently and comprehensively reviewed (42, 43). Instead, we focus on the following six viruses listed in Table 1 that cause acute severe maternal disease, reviewing the innate immune mechanisms and viral evasion strategies that contribute to their effects in pregnancy.

**Table 1:** RNA viruses that cause severe disease in pregnancy.

**Virus**	**Family**	**Genome**	**Global burden of disease**	**Overall case-fatality rate (CFR)**	**Case-fatality rate (CFR) in pregnancy**
Lassa virus	*Arenaviridae*	ssRNA	>500,000 cases annually, endemic in West Africa (44)	1% (44)	33.7% (29)
Ebola virus	*Filoviridae*	ssRNA	>28,000 cases during the 2013–2016 epidemic in West Africa (44)	45–90% (44)	84.3% (45)
Dengue virus	*Flaviviridae*	ssRNA	390 million cases annually (46)	1.1% (47)	3% (48)
Hepatitis E virus	*Hepeviridae*	ssRNA	20 million cases annually (49)	0.2–4% (42)	26% (28)
Pandemic influenza	*Orthomyxoviridae*	ssRNA	1918: >500 million cases 1957: 2 million cases 2009: 1.6 million cases	1918: 2.5% (50) 1957: 0.1% 2009: 2.5% (27)	1918: 27–50% (34, 51) 1957: 30–50%^*^ 2009: 1.7–11% (27, 36, 52–54)
Novel coronavirus infections: - SARS-CoV - MERS-CoV - SARS-CoV-2	*Coronaviridae*	ssRNA	SARS: 8,437 cases 2002–03 MERS: 2,494 cases 2012–13 COVID-19: >21 million cases 2019–20	SARS: 11% (55) MERS: 34% (55) COVID-19: 1.3–4.2% (56)	SARS: 30–40% (57, 58) MERS: 54% (59) COVID-19: 1.2% (60)

*SARS-CoV, severe acute respiratory syndrome coronavirus; MERS-CoV, Middle East respiratory syndrome coronavirus; COVID-19, syndrome resulting from acute infection with novel coronavirus SARS-CoV-2; ssRNA, single-stranded ribonucleic acid*.

* *Maternal mortality in the 1957 “Asian” influenza pandemic is not well described, but half the women of reproductive age (15–44 years) who died were pregnant and the disease became the leading cause of maternal death in Minnesota (35, 61)*.

## Decidual Innate Immunity in Pregnancy: At the Frontier of Maternal-Fetal Tolerance and Infection

**Figure 1:**
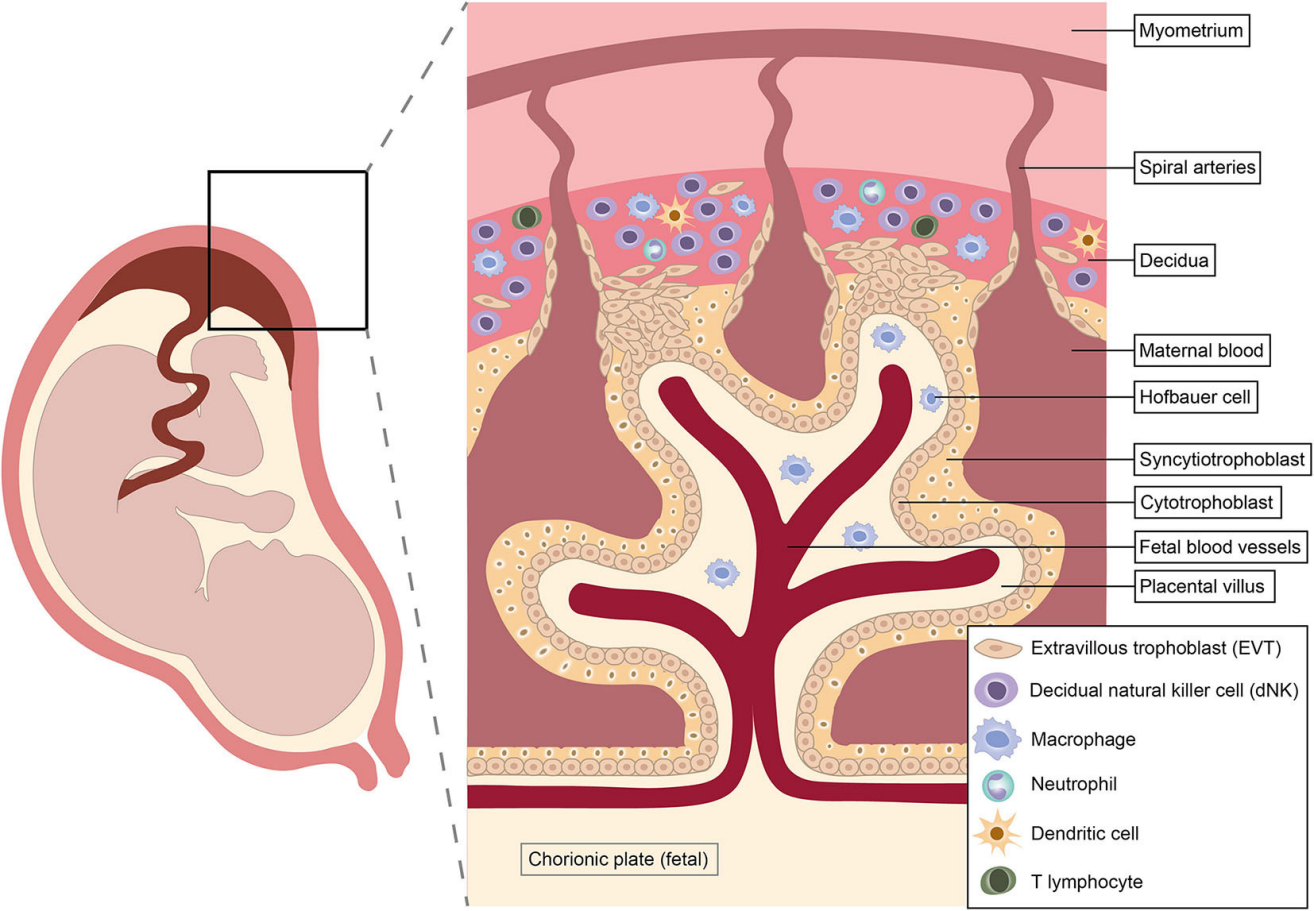
Architecture and immunological repertoire of the maternal-fetal interface. Immune cells shown reflect approximate abundances of different populations in the first and second trimesters of pregnancy. Natural killer cells predominate in early pregnancy, but disappear from mid-gestation onwards (62, 66).

Pregnancy is a unique immunological state. Alterations in systemic maternal immunity and cellular dialogue at the maternal-fetal interface combine to maintain tolerance of the fetal allograft while simultaneously preserving the ability to respond to infection. Shortly after conception, rising progesterone levels trigger decidualization, the transformation of the endometrium into a specialized tissue that promotes implantation of the blastocyst (62). Intensive study of the cellular composition of the decidua over recent years has identified diverse immune cell populations, including natural killer (NK) cells, macrophages, dendritic cells (DC) and T cells (63–65) (Figure 1). Interactions between these decidual immune cells and the invading fetal extravillous trophoblast exert a critical influence on subsequent placentation, fetal growth and pregnancy outcome (23, 25, 67–69).

The unique non-classical human leukocyte antigen (HLA) class I molecule HLA-G is exclusively expressed on extravillous trophoblast (EVT) (70, 71). Invading EVT comes into direct contact with maternal cells when it infiltrates through the decidua into the myometrium, remodeling maternal spiral arteries into dilated low-resistance channels that maximize blood flow to the developing feto-placental unit (42).

The EVT is capable of both immune evasion *and* induction of tolerance due to its unique HLA expression profile, consisting of only the class I molecules HLA-C, HLA-E, and HLA-G. Trophoblast HLA-G undergoes high-affinity binding with leukocyte immunoglobulin-like receptor B1 (LILRB1), an inhibitory receptor widely expressed on decidual antigen-presenting cells. This interaction modulates decidual DC signaling, suppresses production of pro-inflammatory cytokines and inhibits proliferation of maternal T cells. HLA-G therefore provides a critical tolerogenic signal at the maternal-fetal interface (72–74).

The role of decidual innate immune cells in defense against infection is an emerging and rapidly evolving field. Current knowledge is summarized in a recent review by Yockey et al. (75) and relevant aspects of the decidual innate immune response to viral infection will be discussed in detail below. Despite significant advances, our understanding of the recalibration of the innate-adaptive equilibrium in pregnancy remains incomplete. This is due to the practical and ethical difficulties of obtaining decidual tissue samples and the uncertain correlation between peripheral and decidual immune cell activity (6, 25, 76). Renewed focus on the role of the maternal innate immune system in the apparent conflict between fetal tolerance and robust defense against intracellular pathogens will provide further insights into how these two priorities interact in pregnancy. For the purposes of this review, the term “tolerance” refers to the pregnancy-specific state of tolerance of the semi-allogeneic fetus, rather than to the specific T cell phenomenon.

## Systemic Innate Immunity in Pregnancy

Systemically, there is a global upregulation of innate immune cells and effector mechanisms in normal pregnancy (6, 77). Complement activity increases compared to the non-pregnant state (78, 79) and there is a substantial rise in circulating phagocytes and type I interferon (IFN)-producing plasmacytoid DC with advancing gestational age (9, 80). Longitudinal studies of serial blood samples from pregnant women show specific enhancement of innate pathways that mediate antiviral immunity: for example, IFN-α-induced STAT1 signaling, a critical response to viral challenge, increases throughout gestation in NK cells, monocytes and myeloid DC (6, 81). This state must be finely calibrated: excessive activation can be associated with tissue damage during response to acute viral infection (9, 77, 82, 83) and adverse obstetric outcomes [such as complement overactivity in antiphospholipid syndrome (84, 85)], while an attenuated immune response could predispose to overwhelming infection. Whether maternal susceptibility to RNA viral infections is due to over- or under-activity of the innate immune system is not yet clear; it is likely that some effector mechanisms are upregulated while others are suppressed. Data specific to individual viruses will be discussed below.

Of all the viruses discussed in this review, influenza has received the most scientific attention with regards to its propensity for pregnancy, but precise mechanisms underlying this vulnerability remain uncertain. While some animal models (86, 87) and a few *ex vivo* human studies (82, 83) have determined specific innate immune correlates of the increased severity of influenza in pregnancy, much less is known about the other viruses included in this article. This review will synthesize available knowledge on the nature, magnitude, and timing of innate immune responses to viral infection in pregnancy and how these interact with decidual, hormonal and physiological influences.

## Molecular Mechanisms of Innate Immunity to Viruses

Human antiviral immunity is a two-step process. Non-specific innate mechanisms are activated immediately, predominate during the first 5–7 days of infection, and are then superseded by T and B cell-mediated antigen-specific adaptive responses. Rapid-onset innate responses may be sufficient to eliminate the virus, but if not, they limit replication during the critical temporal gap between onset of viral challenge and development of adaptive virus-specific cytotoxic lymphocytes, reducing the likelihood of disseminated disease (88).

The innate antiviral response is activated when pattern-recognition receptors (PRRs) detect pathogen-associated molecular patterns (PAMPs), particularly viral nucleic acids. Toll-like receptors (TLRs) and RIG-I-like receptors (RLRs), which are expressed on innate cell membranes and throughout the intracellular compartment, are the primary viral sentinels (89). This PRR-PAMP interaction triggers activation of latent transcription factors that upregulate a vast repertoire of antiviral effector proteins: type I interferons, other pro-inflammatory cytokines such as TNF and IL-1β, chemokines and the antimicrobial peptides (e.g., defensins, cathelicidins, surfactant proteins) (88, 90, 91).

### The Interferon Response

**Figure 2:**
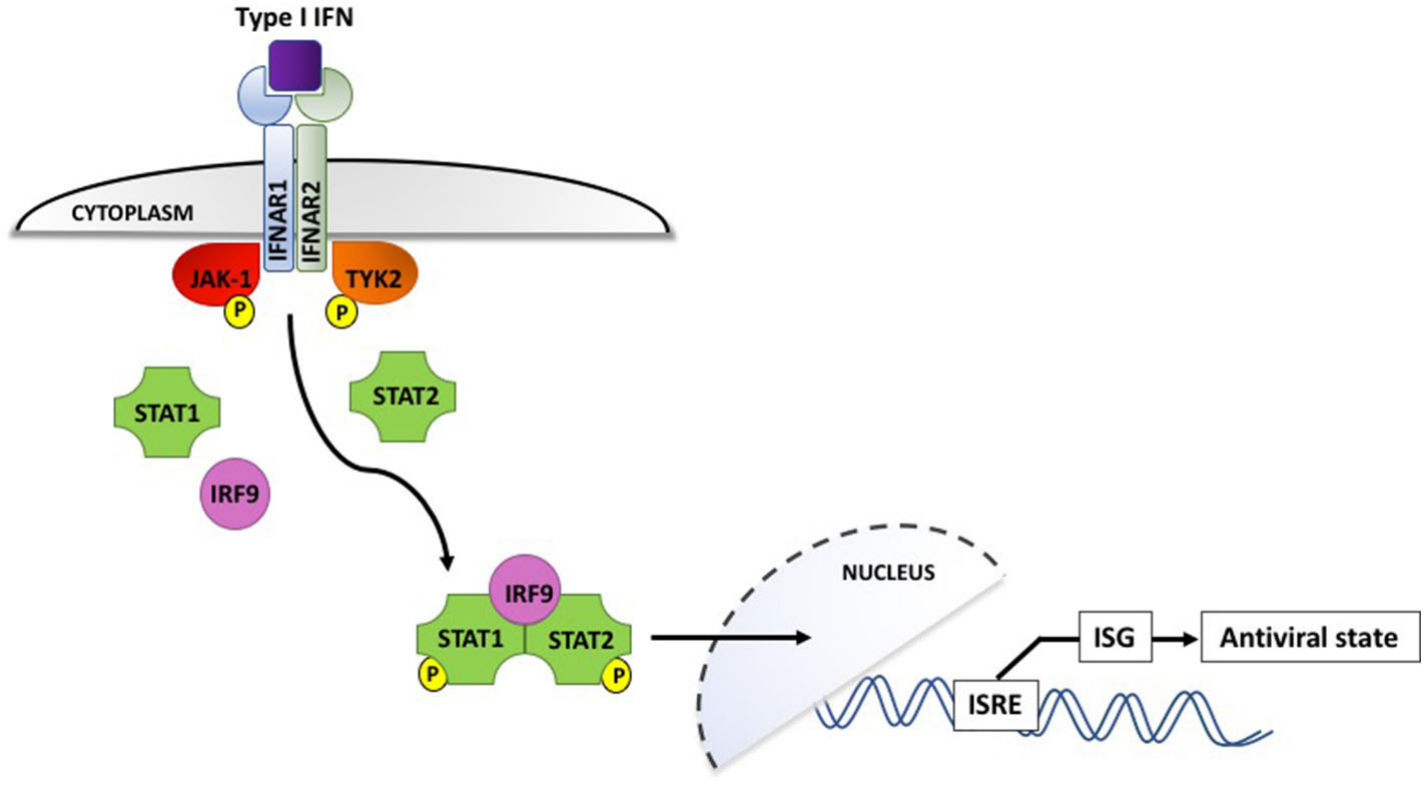
Type I interferon signaling via the IFNAR receptor induces phosphorylation and activation of the JAK-1 and TYK2 tyrosine kinases, which interact with IRF9 to upregulate interferon-stimulated genes and induce an antiviral state in the surrounding cellular microenvironment. IFN, interferon; IFNAR, interferon α/β receptor; IRF9, interferon regulatory factor 9; ISG, interferon-stimulated genes; ISRE, interferon-stimulated response elements; JAK-1, Janus-associated kinase I; STAT1/2, signal transducer and activator of transcription 1/2; TYK2, tyrosine kinase 2. P denotes phosphorylation.

Interferons, which are classified according to their cell surface receptors, are grouped into three types. Type I (including IFN-α and IFN-β) and type II (IFN-γ) are produced by virtually all cells, while the more recently discovered type III (IFN-λ) is produced by epithelial and dendritic cells (92). The production of type I IFNs is the hallmark of effective antiviral immunity. Once secreted by virally infected cells, they act in a paracrine manner to induce IFN-stimulated gene (ISG) expression in neighboring cells, creating an antiviral state in the surrounding environment (shown in Figure 2). IFN binding to the type I IFN receptor, IFNAR, leads to receptor endocytosis and activation of the receptor-associated tyrosine kinases Janus kinase I (JAK-1) and tyrosine kinase 2 (TYK2). These in turn activate transcription factors STAT1 and STAT2, which associate with interferon regulatory factor 9 (IRF9). IRF9 is translocated to the nucleus and triggers IFN-stimulated response elements (IRSEs) to upregulate expression of ISG, the definitive effectors of the antiviral response (91–93). Type III IFN can also induce ISG but their effects appear to be limited to sites of epithelial damage at anatomical barriers, exerting a localized antiviral response that is superseded by potent systemic type I IFNs if the infection is not successfully contained (94).

### Cellular Interactions With RNA Viruses

**Figure 3:**
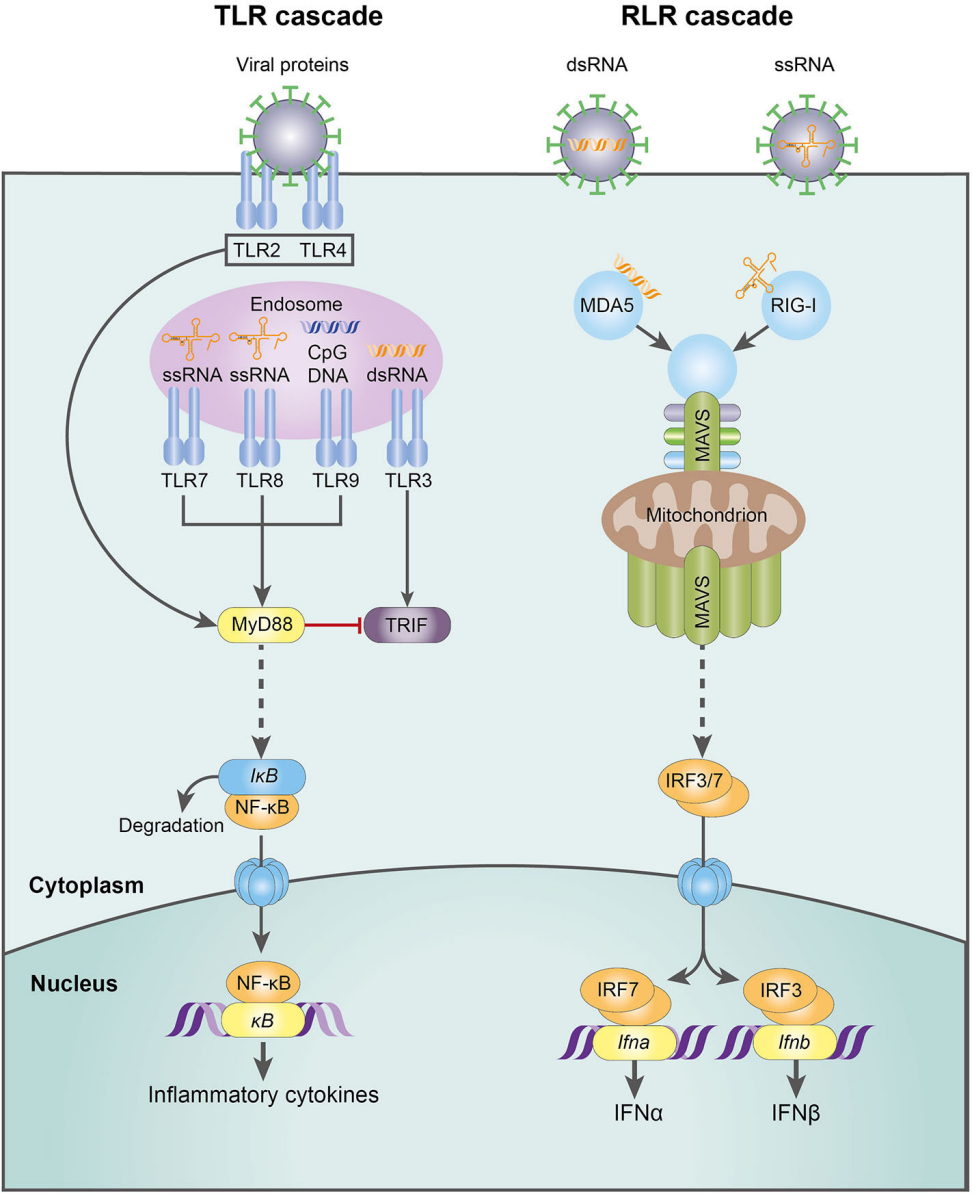
The molecular mechanisms of the TLR- and RLR-mediated innate response to RNA virus infection. CpG DNA, cytosine-guanine oligodeoxynucleotides; dsDNA, double-stranded deoxyribonucleic acid; IFN-α, interferon-α; IFN-β, interferon-β; IkB, inhibitor of NFkB; IRF3, interferon regulatory factor 3; IRF7, interferon regulatory factor 7; MAVS, mitochondrial antiviral-signaling protein; MDA5, melanoma differentiation-associated protein 5; MyD88, myeloid differentiation primary response 88; NFkB, nuclear factor kappa-light-chain-enhancer of activated B cells; RIG-I, retinoic acid-inducible gene I; RLR, RIG-I-like receptor; ssRNA, single-stranded ribonucleic acid; TLR, Toll-like receptor; TRIF, TLR-domain-containing adapter-inducting interferon-β.

RNA viruses, the focus of this review, are detected by a variety of PRRs. Plasmacytoid dendritic cells (pDC) detect viruses via endosomal TLR7-9 and are responsible for the first wave of type I IFN production, releasing large quantities through the MyD88-IRF7 pathway [reviewed in (95)]. In contrast, macrophages and conventional dendritic cells (cDC) sense viral challenge through RLRs, specifically the cytoplasmic helicases RIG-I and MDA-5. These receptors signal via mitochondrial antiviral-signaling protein (MAVS) and IRF3/7, culminating in a second wave of type I IFN production (91, 95) (Figure 3). The emerging role of NK cells in antiviral immunity and pregnancy, recently reviewed in this journal (66), has provoked controversy and is discussed in more detail below.

### Viral Interactions With Programmed Cell Death Pathways

Since viruses depend on host cells for replication, programmed cell death pathways—including apoptosis, necroptosis, and pyroptosis—are a crucial component of antiviral defense. In apoptosis, infected cells undergo an orderly caspase-mediated degradation and are rapidly cleared by surrounding phagocytes (96). This highly regulated disassembly minimizes the release of damage-associated molecular patterns (DAMPs) that could trigger harmful auto-inflammatory responses, but also fails to induce robust antiviral immunity (97).

Necroptosis and pyroptosis differ from apoptosis in that they are powerfully immunogenic. They induce lytic cell death, triggering release of DAMPs and pro-inflammatory cytokines, as detailed in a recent review (98). Although these mechanisms have been implicated in a wide range of autoimmune disorders [reviewed in (99)], they are also a key component of antiviral immunity.

Necroptosis is a caspase-independent process that can be triggered through several different mechanisms, including TNF signaling and TLR detection of viral molecular signatures. These pathways converge on receptor-interacting protein kinase-3 (RIPK3), which phosphorylates and activates the pseudokinase mixed lineage kinase domain-like protein (MLKL). Activated MLKL undergoes conformational changes that expose its pore-forming 4-helical bundle domain, leading to rapid cell lysis (97, 100).

Pyroptosis, a swift and powerfully pro-inflammatory form of programmed cell death, results from activation of the cytosolic NLRP3 inflammasome in virally infected cells. Its caspase-1 effector domain cleaves and activates gasdermin-D, triggering lethal pore formation in the host cell membrane and efflux of pro-inflammatory cytokines (101, 102).

### Complement Antiviral Responses

The complement system is another crucial component of antiviral defense. It bridges the innate-adaptive divide through its diverse roles: opsonization and lytic destruction of pathogens, clearance of apoptotic cells and immune complexes, phagocyte chemotaxis and mast cell activation. A highly conserved cascade of over 50 circulating and membrane-bound protein components, it can be activated through three separate pathways: classical, alternative, and lectin. All converge on the formation of C3 convertases, which cleave C3 into active fragments C3a and C3b. Deposition of C3b on cell or pathogen surfaces triggers formation of the C5 convertase, which splits C5 into C5a and C5b, catalyzing the formation of the membrane attack complex (MAC) that penetrates viral membranes to induce lytic destruction (26, 103–105).

### Regulation of the Antiviral Response

The pro-inflammatory antiviral response is tightly calibrated: inadequate responses lead to overwhelming infection, while excessive activity causes host tissue damage and is associated with autoimmune disease. A variety of inbuilt negative feedback post-translational modification systems orchestrate spatial and temporal regulation of the antiviral response. For example, ubiquitin-specific peptidase 18 (USP18, also known as ISG43) displaces JAK1 from IFNAR to block type I IFN signaling and also deconjugates ISG15 (one of the most potent inhibitors of viral replication) from its target proteins (91, 106–108).

Such mechanisms are not always sufficient to prevent dysregulation of type I IFN activation, which has been implicated in several auto-inflammatory conditions including systemic lupus erythematosus (91, 109), a disease which shows excessive complement deposition. Similarly, excessive complement activation contributes to acute lung injury in mouse and human studies of influenza and novel coronaviruses, through over-production of the anaphylatoxins C3a and C5a (110–112).

The following sections examine the individual cellular components and effector mechanisms of the innate antiviral response that undergo significant adaptations in pregnancy.

## Pregnancy-Induced Modification of Antiviral Immunity

### Antimicrobial Peptides

Antimicrobial peptides (AMP) are small molecules that create a microbicidal shield at mucosal surfaces with high pathogen exposure, such as the small intestine, the renal epithelium, and the chorionic membranes. They are secreted by leukocytes and disrupt pathogen membrane integrity, leading to lytic destruction. Three classes of AMP that contribute to antiviral immunity exhibit differential expression in pregnancy: defensins, cathelicidins, and surfactant proteins.

Human defensins are categorized into three families (α, β, and θ). Alpha-defensins are found in neutrophil granules and Paneth cells while beta-defensins are expressed in epithelial cells, including those of the female reproductive tract, and are upregulated by cytokine secretion in response to pathogen-mediated epithelial injury (113, 114).

During pregnancy, the AMPs human-beta-defensin-1 (HBD-1) and HBD-3 are elevated in the amniotic fluid of women who develop preterm labor with coexistent infection (115, 116). HBD1-3 are also expressed in the trophoblast and decidua, suggesting a critical physiological role for defensins in innate immune defenses at the maternal-fetal interface; they also participate in innate-adaptive crosstalk through chemotaxis, recruiting T cells and immature DC (117).

Like defensins, cathelicidins are small AMP secreted by innate cells and mucosal barriers. The sole human cathelicidin, LL-37, is vitamin D-inducible and promotes wound healing, angiogenesis and clearance of cell debris; it can also regulate macrophage and DC responses to pro-inflammatory stimuli (118). LL-37 expression in first-trimester cervicovaginal secretions is significantly higher in women with bacterial vaginosis and incubation of endocervical epithelial cells with LL-37 *in vitro* induces a pro-inflammatory milieu with enhanced secretion of IL-8 (119). Circulating serum LL-37 is also elevated during pregnancy: LL-37 levels rose consistently in serial samples from Ugandan pregnant women, peaking in the third trimester (120). These studies suggest a role for LL-37 in decidual and systemic pregnancy-specific innate immune defenses.

AMP have been implicated in the innate response to influenza A virus (IAV). LL-37 neutralizes IAV in mouse models by directly damaging viral membranes, whereas surfactant protein-D (SP-D) works by triggering viral aggregation and inhibiting haemagglutinin activity (121–124). In human monocytes, LL-37 and SP-D readily block replication of seasonal IAV, but both had strikingly impaired inhibitory activity against the pandemic H1N1 strain, whereas antiviral activity of a related AMP, H-ficolin, was unchanged (125, 126). The significance of this strain-specific discrepancy in AMP-mediated anti-influenza immunity in pregnancy requires further clarification.

### Toll-Like Receptors

The placenta expresses the full repertoire of human TLRs (TLR1-10) (127). Of these, TLR3, TLR7, TLR8, and TLR9 contribute to antiviral immunity: TLR3 binds to double-stranded viral RNA; TLR7 and 8 detect single-stranded RNA viruses; and TLR9, which recognizes unmethylated cytosine-guanine (CpG) motifs in bacterial genomes, can also respond to herpesvirus infection. Antiviral PRR signaling mostly converges on the canonical MyD88 pathway, stimulating activation of NFκB and production of pro-inflammatory cytokines (128, 129). The exceptions are TLR3 and RIG-I, which signal through an alternative MyD88-independent pathway that uses an adapter protein, TRIF, to generate large amounts of IFN-β (130).

TLR expression at the maternal-fetal interface exhibits both temporal and tissue-specific fluctuations in expression levels and functionality (131–134). This suggests a potential contribution to the observed differences in severity of fetal and maternal viral infections in different trimesters (42, 44, 48, 66, 135). In human trophoblast, TLR3 is highly abundant in the first trimester and forms a defensive barrier along the cytotrophoblast with TLR2 and TLR4 (136). TLR3 has dual roles at the placental interface, inhibiting viral replication to protect the developing fetus from vertical transmission but also promoting tolerance through release of indoleamine 2,3-dioxygenase (IDO) (137–139). In the decidua, Duriez and colleagues have demonstrated differential expression of the four antiviral TLRs in decidual macrophages and NK cells, with each cell type producing a distinct cytokine signature in response to TLR7/8 ligation (140). These TLR-mediated responses to viral challenge are at the frontier of the critical balance between tolerance and immunity, which ultimately dictates whether or not a pregnancy will be successful (141).

### Complement

In pregnancy, complement activity is increased systemically but suppressed at the maternal-fetal interface (26, 142). While some complement components contribute to normal placentation [C1q, for example, promotes adequate EVT invasion and remodeling of maternal spiral arteries (143, 144)], the majority must be inhibited to ensure successful pregnancy. Synergistic action of regulatory molecules at the placenta impairs formation of the classical and alternative C3 convertases, preventing downstream activation of the C5 convertase and the MAC (104). Failure to suppress these pathways is associated with a wide range of adverse obstetric outcomes, including recurrent miscarriage, fetal growth restriction, preterm birth and pre-eclampsia (26, 104, 145).

The complement system mediates neutralizing antiviral immunity through multiple effector mechanisms, including:

1. MAC-induced pore formation in viral envelopes, leading to lytic destruction;
2. MAC-independent virolysis through deposition of complement components on non-enveloped viruses or direct binding to MBL;
3. Opsonization of virions: this induces aggregation, decreasing the total infectious burden for the host, and may be followed by phagocytosis (103, 146, 147).

**Figure 4:**
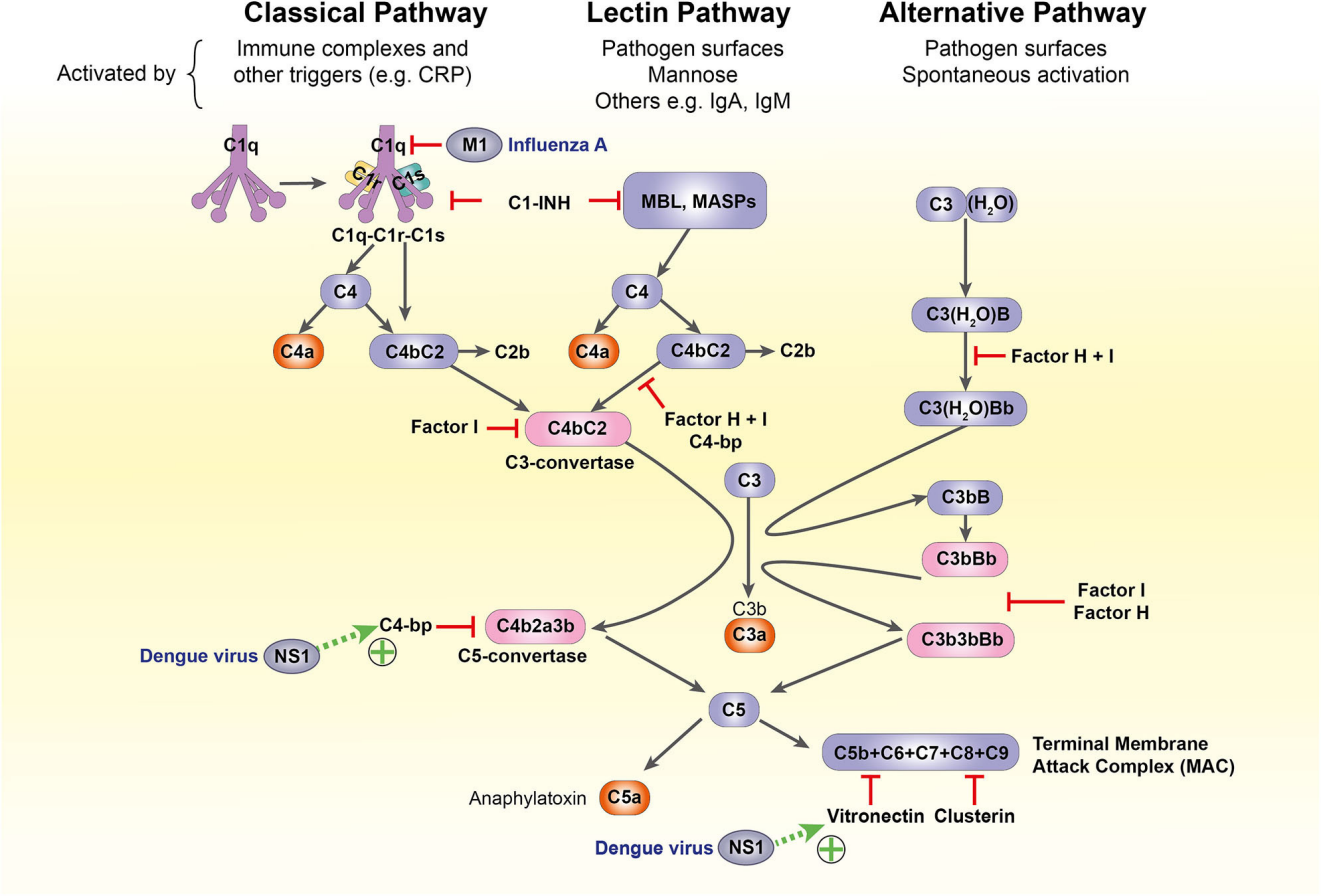
The complement system and its subversion by dengue and influenza A viruses. C1-INH, C1-inhibitor; C4-bp, C4-binding protein; CRP, C-reactive protein; IgA/IgM, immunoglobulin A/M; M1, influenza virus matrix protein 1; MASP, MBL-associated serine protease; MBL, mannose-binding lectin; NS1, non-structural protein 1.

However, the diversity of methods for complement-mediated viral attack is mirrored and in some cases surpassed by viral evasion mechanisms, which vary widely according to species and virion structure. For example, Dengue virus non-structural protein 1 is extruded from infected cells and recruits terminal complement inhibitors C4b-binding protein and vitronectin to the cell surface, inhibiting MAC assembly (148, 149); the influenza virus matrix protein 1 inhibits C1q-mediated activation of the classical pathway (150) (Figure 4). These mechanisms have not been characterized in pregnancy and a complete discussion of complement subversion by viruses is beyond the scope of this article, but further detail is available in a recent review (103).

### Cellular Innate Immunity

Various cellular mechanisms are exploited by viral infection, a number of which are altered during pregnancy.

#### Innate Lymphoid Cells

Innate lymphoid cells (ILC), which account for up to 70% of all leukocytes present in first-trimester decidua (62), originate from the common lymphoid progenitor and bridge the innate-adaptive divide. Like other innate cells, they mount rapid responses to infection, lack antigen-specific receptors, and do not exhibit conventional clonal expansion; like T cells, they can modulate adaptive immune responses through production of specific cytokines and regulation of B cell and DC activity. On this basis, ILC have been described as “innate counterparts” of T cells (151). Their abundance at the maternal-fetal interface suggests pivotal roles in both innate immune defense and normal placental development (152). Table 2 summarizes the classification and functions of decidual ILC:

**Table 2:** The roles of decidual innate lymphoid cell subsets in pregnancy (152–155).

**Subset name**		**Phenotype**	**Greatest abundance**	**Key effectors**	**Roles in the decidua**
ILC1	dNK cells	CD56^bright^ CD16^−^ KIR^++/−^ CD9^+^ Tbet^+^ Eomes^+^	Early pregnancy	Perforin Granzyme B Granulysin Cytokines	Tolerance Trophoblast invasion Tissue remodeling Antiviral immunity
	Non-cytotoxic ILC1	CD56^−^CD127^−^ CD117^−^Tbet^+^ Eomes-	Early pregnancy	IFN-γ	Defense against intracellular bacteria and parasitic infection Allergy
ILC2		CD56^−^CD127^+^ CD161^+^Tbet^−^	Late pregnancy	IL-4, IL-5, IL-13, IL-22	Tolerance Tissue repair Homeostasis
ILC3	NCR^+^	CD127^+^CD117^+^NCR^+^	Early pregnancy	IL-8, IL-22, GM-CSF	Tissue remodeling Neutrophil recruitment and activation
	NCR^−^	CD127^+^CD117^+^NCR^−^	Early pregnancy	IL-17A, TNFα	Renewal of NCR^+^ population through ILC3 plasticity
	LTi-like	Defined by RORγt expression	Early pregnancy	IL-17A, TNFα	Tissue remodeling Recruitment of other immune cells

*dNK cells, decidual natural killer cells; Eomes, eomesodermin; GM-CSF, granulocyte-macrophage colony-stimulating factor; IFN, interferon; IL-, interleukin-; ILC, innate lymphoid cells; KIR, killer-cell immunoglobulin-like receptors; LTi, lymphoid tissue inducer, NCR, natural cytotoxic receptor; RORγt, RAR-related orphan gamma receptor; T-bet, T-box expressed in T cells; TNF, tumor necrosis factor*.

Given the recent discovery of ILC, data on their antiviral properties are still accumulating. In the liver, rapid ILC1-mediated IFN-γ production is essential for early suppression of cytomegalovirus (CMV) viremia (156); in the lungs, production of amphiregulin by ILC2 is critical for restoration of epithelial integrity and lung function following influenza infection, while excessive remodeling can predispose to allergy (157, 158). In pregnancy, investigations have focused on NK cells, both peripheral and decidual.

#### Peripheral Blood Natural Killer Cells

The majority (90%) of peripheral blood NK (pbNK) cells exhibit a predominantly cytotoxic CD56^dim^ CD16^+^ phenotype, while the remaining 10% constitute the main cytokine-producing CD56^bright^ CD16^−^ subset (25). Although not antigen-specific, NK cells can utilize the Fc-receptor CD16 to target cells for destruction. Aside from CD16, NK cells integrate signals from an array of germline-encoded activating and inhibitory receptors. Inhibitory signals transmitted through interaction with the ubiquitously expressed HLA class I and the non-classical HLA-E dominate in the healthy human. This balance can be shifted in the case of diseases such as cancer, in which HLA-I and HLA-E may be downregulated, and instead ligands for activating receptors can be induced on transformed cells (e.g., NKG2D ligands) (159).

NK cell receptors can distinguish between “self” and “non-self” through detection of HLA class I and related molecules: their absence can be sensed and triggers degranulation and release of cytotoxic components. Through the same mechanism, viruses, which downregulate HLA-I expression in the cells they infect, become more susceptible to NK-mediated cytotoxicity (76). In a recent study, Le Gars et al. used mass cytometry to compare the responses of isolated pbNK cells from pregnant and non-pregnant women to *ex vivo* challenge with IAV. In the pregnant cohort, production of IFN-γ by both pbNK subsets was significantly upregulated, as was their capacity to kill influenza-infected monocytes (83). Although robust NK cell activity is usually important for viral clearance, this pregnancy-specific NK enhancement may actually be detrimental: IL-15-deplete mice, who cannot mount NK-mediated responses to influenza, show significantly improved survival compared to controls (160).

#### Decidual Natural Killer Cells

Decidual NK (dNK) cells are a unique population of CD56^bright^ CD16^−^ KIR^++/−^ cells that expand in the peri-implantation window and remain highly abundant until the end of the second trimester, after which their numbers gradually diminish (66). Despite some phenotypic overlap with pbNK, dNK exhibit a unique set of surface markers and are functionally distinct: their cytotoxic capacity toward allogeneic “non-self” cells, namely the trophoblast with which they are in direct contact, is completely abrogated (62). The lack of dNK cytotoxicity was initially attributed to an attenuation of cytotoxic granule components. However, this has proved wrong: paradoxically, dNK in fact possess equivalent or higher levels of granzyme B, perforin, and granulysin than pbNK (30, 62). The issue is one of translocation: dNK fail to polarize cytotoxic granules to the immunological synapse with target non-self-cells (161). Similarly, it was presumed that these non-cytotoxic dNK would lack the ability to kill virally infected cells. This was disproved when Siewiera et al. demonstrated that dNK isolated from first-trimester decidua rapidly developed into cytotoxic effectors on exposure to CMV-infected autologous decidual fibroblasts, efficiently mobilizing cytolytic apparatus to the immunological synapse and infiltrating CMV^+^ trophoblastic tissue in culture (31). Importantly, this cytotoxic effect was lost when experiments were repeated with CMV-positive primary EVT, which dNK were unable to kill (162). Whether a similar pattern occurs with other viruses currently remains unproven, and observations specific to CMV should not be extrapolated to other infections.

These findings have led to renewed interest in NK-mediated antiviral immunity and the capacity of viruses to subvert molecular mechanisms of NK activity. In the case of CMV, viral infection can exert unique selective pressures on the pbNK cell compartment, influencing KIR acquisition and triggering clonal expansion of CD57^+^ populations expressing specific repertoires of the C-type lectin NKG2C (163, 164). However, it is important to emphasize that currently these data refer only to CMV and equivalent effects with other viruses have not been demonstrated. Similarly, expansions of this kind have not been observed among uterine NK cells and it remains uncertain whether the pbNK cell pool (where such proliferation may exist) influences the endometrial or decidual NK cell composition (164). Whether this imprint on NK cells induces long-term immunological memory against repeated viral challenge has proved controversial in humans, where peripheral and lymphoid tissue-resident NK appear to develop memory, as demonstrated in studies of CMV (165), hantavirus (166), and varicella-zoster virus (167). However, an equivalent effect in the uterus remains to be clarified. Overall, the precise role of dNK in antiviral immunity remains controversial and largely unknown.

#### Neutrophils

Pregnancy induces a physiological neutrophilia both systemically and at the decidua. In pregnant women, neutrophils account for up to 95% of peripheral blood leukocytes, compared to 50–70% outside pregnancy (80, 168). This reflects a state of enhanced innate immune vigilance against infection: neutrophils, the most abundant of all human phagocytes, are recruited to sites of infection or injury by chemokines such as complement C5a and form the first line of defense against microbial challenge. Engulfed micro-organisms are internalized in phagolysosomes and destroyed using a wide range of effector mechanisms including release of serine proteases (e.g., neutrophil elastase, cathepsin G) and production of reactive oxygen species (141, 169). Pathogens that evade phagocytosis can be controlled by the release of neutrophil extracellular traps (NET), large reticular structures containing cytotoxic granule proteins and AMP that sequester and neutralize microbes (170). While the role of NET in defense against bacterial and fungal infections is well-established, their contribution to antiviral immunity has been more difficult to ascertain (171, 172). NET formation may actually be detrimental in viral infections (173), with recent data showing that high levels of NET production exacerbate acute lung injury in influenza (174, 175) and can worsen prognosis in dengue virus infection (176).

The origins of this pregnancy-specific neutrophilia are incompletely understood but are likely to include progesterone-induced upregulation of granulocyte-macrophage colony-stimulating factor (GM-CSF) (177, 178). Mass cytometry analysis of circulating neutrophils in pregnancy by Aghaeepour et al. revealed not only increased abundance but also a hyper-activated state, with progressively enhanced sensitivity to pro-inflammatory stimuli including IL-6 and TNFa (6). However, the same group have also demonstrated a linear increase in “immature-like neutrophil signatures” (denoted by CD10 and CD15 expression) with advancing gestational age, which they suggest could contribute to the increased mortality observed in acute influenza and hepatitis E infections during late pregnancy (179).

Chemokine synthesis in the decidua leads to an influx of innate immune cells. Contact with invading trophoblast during implantation stimulates production of neutrophil chemo-attractants IL-8, CXCL1, and CXCL2 by decidual stromal cells, a process potentiated by the addition of progesterone *in vitro* (180, 181). However, the existence of a decidual neutrophil population remains controversial. Although one study from 2014 identified neutrophils in second-trimester decidua (182), this has not proved reproducible in subsequent single-cell reconstructions of the maternal-fetal interface (183, 184).

#### Macrophages

Monocytes are myeloid leukocytes that are released from the bone marrow, mature in the peripheral circulation and infiltrate into tissues, where they can differentiate into specialized populations of macrophages or myeloid DC. As with neutrophils, the monocyte-macrophage lineage expands both systemically and in the uterus during pregnancy (24, 185): macrophages account for up to 25% of the decidual leukocyte population and are outnumbered only by NK cells (80, 141). Macrophages have long been recognized as key phagocytic immune sentinels at mucosal surfaces. At the maternal-fetal interface their roles include antigen presentation to adaptive immune cells, promotion of tolerance through clearance of apoptotic trophoblast debris, secretion of pro-angiogenic factors and direct participation in spiral artery remodeling through the phagocytosis of maternal vascular smooth muscle cells (24).

The diverse functions of macrophages arise from their capacity for polarization into two antagonistic phenotypes, M1 (pro-inflammatory) and M2 (anti-inflammatory) (186). In normal pregnancy, the M1/M2 equilibrium is in constant flux, with M1 predominating during early trophoblast invasion, a shift toward tolerogenic M2 in the second and third trimesters, and a reversion to M1 at the onset of parturition (24). Differential TLR activity in decidual macrophages may contribute to protection of both mother and fetus against infection: stimulation of decidual macrophages with TLR2-4 and 7/8 agonists restricts viral replication and leads to overproduction of IL-10, an anti-inflammatory cytokine that suppresses trophoblast TLR signaling (140, 187).

Pregnancy-specific adaptations in the M1/M2 balance have also been implicated in the response to systemic viral infection. A study comparing the response of peripheral blood monocytes from pregnant and non-pregnant women to *in vitro* challenge with IAV found that the pro-inflammatory response was exaggerated in pregnant women, although trimester-specific effects were not reported (82). In a mouse model of pandemic H1N1 IAV in pregnancy, mice infected in mid-gestation exhibited higher clinical severity scores and a strong phenotypic shift toward the M2 phenotype in alveolar macrophages obtained from broncho-alveolar lavage samples, compared to those from non-pregnant matched females (87).

#### Dendritic Cells

Like macrophages, DC are critical for innate-adaptive crosstalk. DC can be broadly divided into two subsets:

1. Myeloid DC (mDC, CD14^−^CD11c^+^), which exist in an immature state in the spleen and lymph nodes and can migrate into various tissues where they are associated with Th1-type pro-inflammatory responses
2. Plasmacytoid DC (pDC, CD123^+^CD11c^−^), which reside in non-lymphoid tissues and induce Th2-type responses and proliferation of Tregs (25).

Circulating mDC from pregnant women demonstrate a skewed cytokine response to inflammatory stimuli, with increased production of IL-10, programmed death-ligand 1 and CD200, favoring Th2 and Treg polarization over Th1 (6, 188). DC of myeloid origin are recruited into the decidua from a variety of progenitor populations. Decidual DC (dDC) account for <2% of decidual leukocytes, are a highly proliferative and relatively immature population, and are phenotypically distinct from peripheral DC: they display a universal reduction in expression of T cell costimulatory molecules and thus fail to induce specific T cell responses *in vitro* (189). dDC help to prevent fetal rejection by promoting tolerogenic responses to endocytosed trophoblast antigens, recruit NK cells through production of IL-15 and potentiate decidual angiogenesis via close dialogue with decidual stromal cells (141).

The integral role of pDC in the innate antiviral response to RNA viruses, which centers on their unrivaled capacity for type I IFN production (95), may be further upregulated in pregnancy. In a study comparing pDC isolated from pregnant and non-pregnant women, pDC from pregnant women were more abundant at baseline and demonstrated increased chemokine production (including IFN-inducible protein 10 and CCL4) in response to IAV challenge. The authors note that excessive chemokine release is associated with higher disease severity and mortality from influenza A *in vivo* (190, 191) and suggest that the exaggerated pDC response they observed could contribute to the disproportionately high fatality rates of influenza in pregnancy (82).

#### Decidual Stromal Cells

Trophoblast invasion induces dramatic remodeling of the uterine mucosa, transforming the glands, arteries and stroma of the late-secretory-phase endometrium in a progesterone-dependent process known as decidualization (62, 164, 192). The specialized decidual stromal cells (DSC) generated as a result are fundamental for the establishment and homeostasis of the decidual immune system. In particular, DSC are in constant dialogue with dNK and exert critical regulatory influence over their various functions. Progesterone-induced IL-15 production by DSC is essential for the early expansion of dNK (193) and potentiates their cytotoxic degranulation in response to virally infected cells (194). Conversely, DSC-derived IL-33 inhibits dNK perforin and granzyme A synthesis, reduces their cytotoxic capacity and shifts their cytokine effector profile toward the immunosuppressive IL-4, IL-10, and IL-13 (195–197). The observation that T cell chemokine genes are epigenetically silenced in DSC highlights their role in maintaining a tolerogenic environment (12); indeed, they have even shown promise as a novel therapy for acute and chronic graft-versus-host disease in allogeneic stem cell transplant patients (198, 199). Their roles in viral infection remain poorly defined, but recent data show that murine DSC undergo necroptosis in response to transfection with a synthetic analog of viral RNA (200).

It is therefore clear that DSC, instead of merely functioning as a cellular scaffold that hosts the decidual immune system, are vital contributors to its key functions of simultaneous fetal tolerance and immune defense.

## Acute Viral Infections That Cause Severe Maternal Disease

### Lassa Virus

Lassa virus (LASV) is an enveloped ssRNA arenavirus endemic to West Africa that is acquired through contact with infected rodents (*Mastomys natalensis*, the multi-mammate rat). It causes regular seasonal outbreaks of Lassa fever, responsible for over 500,000 cases and 5,000 deaths per year. In the general population:

- 80% of patients will be asymptomatic or experience a mild non-specific illness;
- 15–20% require hospital admission due to severe disease (which may manifest as mucosal hemorrhage, hepatitis or multi-organ failure);
- Up to 29% will develop sensorineural hearing loss during their recovery;
- 1% will die (44, 201, 202).

However, these figures are strikingly altered in pregnancy. Cohort studies have consistently estimated maternal mortality rates as disproportionately high: 7% in the first two trimesters, rising as high as 87% in the third trimester, with fetal loss rate reported between 75 and 100% (44, 201, 203). The first systematic review of Lassa fever outcomes in pregnancy, published this year, identified just seven studies eligible for inclusion in the meta-analysis. This incorporated a total of 236 women in whom the absolute risk of maternal death was 33.7%, the fetal CFR was 61.5% and the neonatal CFR was 30.2%. Pregnancy conferred a 2.86-fold increase in risk of death (95% confidence interval 1.77–4.63) compared to non-pregnant women of reproductive age (29).

This disparity “underscores the need to prioritize pregnancy” in studies of the immune response to LASV (29). Currently, however, our understanding is almost entirely limited to data from animal models or cultured human cells (29, 204). These have provided insights into the key components of the innate immune response to LASV. The importance of the type I IFN response to LASV has been demonstrated in a non-human primate model: animals who survived viral challenge generated a robust IFN-α response shortly after inoculation, whereas those who died failed to upregulate IFN-α until the terminal phase of illness (205). Given that the type I IFN response to other RNA viruses such as influenza is less effective in pregnancy (206), it is possible that a similar attenuation in the case of LASV infection contributes to the higher mortality in pregnant women.

LASV has also been shown to directly antagonize the type I IFN response through its Z matrix protein, which is common to all arenaviruses, and its nucleoprotein (NP). LASV-NP possesses a unique exonuclease domain that degrades viral dsDNA—usually a potent ligand for RLR—to prevent type I IFN production (207).

In cultured human cells, LASV replicates in DC and macrophages but is remarkably adept at concealing its presence: it does not activate the host cells, induce apoptosis, alter their cell surface expression profile or trigger antigen presentation to T cells. This creates immune-privileged reservoirs that facilitate unchecked LASV replication in early infection, with subsequent systemic spread when the infected cells enter draining lymph nodes (202). It may also be a critical determinant of LASV's pathogenicity—in contrast, infection of macrophages and mDC with Mopeia virus, which shares 75% sequence homology with LASV but is non-pathogenic, leads to rapid activation of both cell types with upregulation of T cell costimulatory molecules and production of pro-inflammatory cytokines including type I IFNs and IL-6 (208–211).

The fact that LASV uses elaborate evasion mechanisms to avoid activation of innate immunity, and that this capacity correlates directly with suppression of adaptive responses, reinforces the role of the innate immune system in defense against Lassa fever. However, no single animal or human study to date has focused specifically on innate anti-LASV responses in pregnancy. The question of why pregnant women experience such high mortality from this disease therefore remains unsolved and should be urgently prioritized in future research.

### Ebola Virus

Ebola virus (EBOV) caused over 11,000 deaths in the 2013–2016 outbreak in West Africa and had catastrophic implications for the region's already fragile public health infrastructure. Cases of Ebola virus disease (EVD) were concentrated in Guinea, Sierra Leone and Liberia, which have among the highest maternal mortality ratios in the world, and it is estimated that at least 100 pregnant women died (212–214).

EBOV causes abrupt onset of a febrile illness that can progress to profuse diarrhea, hemorrhage, meningo-encephalitis and hepatic or renal failure (44). Overall CFR is high (45–90%) and if acquired in pregnancy it is almost always lethal for the fetus (213, 215). However, a recent synthesis of available studies demonstrated no significant difference between mortality rates in pregnant vs. non-pregnant women (214). While this may be the case, caution is required on two counts: firstly, as the authors acknowledge, their analysis is limited by the small size, retrospective nature and considerable heterogeneity of included studies; and secondly, while pregnant women may not be intrinsically more vulnerable to EVD, the fact that they are often carers for sick relatives and make frequent visits to health facilities places them at increased risk of acquiring the disease (212).

Like LASV, EBOV targets myeloid DC and macrophages for entry and replication. Both viruses arrest mDC in an immature state that is permissive for viral replication, but their effect on macrophages is different: unlike LASV, EBOV does activate macrophages (44, 216). This allows them to present antigen to T cells, meaning that adaptive responses are at least partially activated in EVD: paradoxically, rather than being protective, this may exacerbate the strong and rapid upregulation of both innate and adaptive immunity that is characteristic of fatal disease (217).

The role of the IFN response in EVD has proved difficult to elucidate. On one hand, EBOV, like LASV, exhibits complex mechanisms that specifically counteract IFN: its VP35 protein contains an inhibitory domain that can scavenge viral dsRNA to prevent it from binding to RIG-I receptors (218) and its VP24 protein blocks nuclear accumulation of phosphorylated STAT1, a critical transcription factor in IFN signaling (219). On the other hand, certain ISGs such as tetherin have been found to actively suppress EBOV replication (220) and the IFN response is massively exaggerated in patients who die of EVD compared to those who survive, suggesting a contribution of host inflammatory response to disease similar to that seen in influenza (217, 221).

The West African epidemic revealed an unexpected tropism for the placenta: women who survived EVD in early pregnancy with complete resolution of viremia and no fetal loss were found to have unusually high rates of miscarriage and stillbirth weeks or months later, with abundant EBOV RNA detected in placental and fetal tissues (222, 223). Immuno-histochemistry analysis of placentas from EBOV-positive mothers shows accumulation of EBOV antigen within the intervillous space, where it co-localizes with a population of atypical maternal macrophages, and in extravillous trophoblast (224). This may reflect shared dependence on specific endocytic mechanisms: EBOV entry into target cells depends on the Niemann-Pick cholesterol transporter protein Niemann-Pick C1 (NPC-1), a protein that is also expressed on the syncytiotrophoblast (225, 226). These findings raise the suspicion of an EBOV predilection for pregnancy, even if the maternal death rate is comparable to that of the non-pregnant population, and support a rationale for close follow-up of female survivors (214).

### Dengue Virus

The arboviruses are a group of over 100 arthropod-borne RNA viruses including dengue, Zika, Chikungunya, and yellow fever viruses. They constitute a major threat to global public health: 90% of pregnant women live in areas with either endemic or epidemic transmission of arboviruses (47).

Dengue, a spherical enveloped flavivirus with four ssRNA serotypes (DENV1-4), is the most abundant of the arboviruses and occupies two separate environmental niches: a sylvatic cycle, in which DENV circulates between arthropod vectors and non-human primate reservoirs, and an urban cycle, in which humans and *Aedes* mosquitoes are the only hosts. Following inoculation, the majority of people remain asymptomatic, but a minority will develop an acute febrile illness accompanied by severe headache, retro-orbital pain, arthralgia, and a rash. Approximately 1% develop severe dengue, a potentially lethal manifestation of disease that results from a sudden increase in systemic vascular permeability and can cause shock, profound thrombocytopenia, hemorrhage, and multi-organ failure. Mortality in severe dengue can be reduced from over 20 to <1% with good supportive care, but there are no specific antiviral therapies with proven benefit (227).

Quantifying the risks associated with DENV infection in pregnancy has proved particularly challenging due to the high proportion of asymptomatic cases, the lack of large prospective studies with comparison groups, the inevitable reporting bias in small case series and the inherent difficulties in accurate diagnosis of acute dengue fever (dengue IgM cross-reacts with other flaviviruses and coinfections are common) (48). Machado and colleagues were the first to definitively establish the association between pregnancy and severe dengue. They analyzed all 151,604 cases of suspected DENV in Rio de Janeiro from 2007 to 2008, compared outcomes in 99 pregnant women to 447 matched non-pregnant women of reproductive age, and found an increased risk of severe dengue in the pregnant women (odds ratio 3.38) with a trend toward higher mortality (3 vs. 1.1%) (47). A 2018 study added to this by investigating correlates of severe maternal disease in French Guiana: they showed an 8.6-fold increase in the risk of postpartum hemorrhage in the presence of severe dengue (228). Whether or not dengue increases the risk of adverse fetal and perinatal outcomes (specifically miscarriage, stillbirth, preterm birth, and low birthweight) remains controversial, with two meta-analyses from 2016 and 2017 reporting conflicting results (229, 230).

The origins of this increased susceptibility to severe dengue in pregnancy remain unknown but are likely to include placental tropism, innate immune adaptations and the physiological increase in vascular permeability that occurs in normal pregnancy, which may both delay diagnosis and exacerbate severe dengue (40). An immunohistochemical analysis of placentas from women with dengue in pregnancy showed accumulation of DENV antigen in the trophoblast and decidua in 92% (22/24) of cases. Microscopy revealed hypoxic changes (including villous edema and infarction) and an unusual observation of sickled erythrocytes in the intervillous space—this occurred despite no history of maternal sickle cell disease and was correlated with maternal death, suggesting that the virus may be able to influence erythrocyte biology in pregnancy (231, 232).

DENV can infect and deplete human megakaryocytes, suppressing their capacity to produce platelets and causing dengue-induced thrombocytopenia. Campbell et al. found that DENV leads to marked upregulation of interferon-induced transmembrane protein 3 (IFITM3) on platelets with corresponding release of type I IFNs, and that the highest levels of IFITM3 expression correlated with mildest disease (233, 234). These mechanisms are yet to be studied in pregnancy, but it is conceivable that enhanced DENV-mediated megakaryocyte depletion or a failure to upregulate platelet IFITM3 could contribute to the higher rates of hemorrhagic complications during pregnancy.

Type I IFN production is central to the innate anti-DENV response, although no specific studies have been conducted in pregnancy. DENV triggers type I IFNs through binding with various PRRs (RIG-I, endosomal TLR3, and endosomal TLR7) and is detected by the mannose-binding lectin complex. This leads to deposition of Cb4 and C2a on the virion surface, formation of the C3 convertase and activation of complement-mediated virolysis, although the virus can subvert this through its non-structural protein NS1 as described above. RNA interference and activation of apoptosis in infected cells are also important contributors to DENV defense, as reviewed by Uno and Ross (96). NS1 also appears to be a critical regulator of the DC and NK-mediated response to dengue infection. Sentinel cutaneous DC detect invading DENV and rapidly recruit NK cells through contact-dependent IFN-mediated upregulation of adhesion molecules, and the NK are key for viral suppression: in humanized mice, depletion of NK cells exacerbates DENV viremia and thrombocytopenia (235).

Although mechanistic insights into DENV pathogenicity with plausible relevance to pregnancy continue to emerge—for example, the observation that vitamin D supplementation reduces cultured human DC susceptibility to DENV2 through downregulation of TLR3, TLR7, and TLR9 signaling (236)—these findings cannot be extrapolated to the pregnant population and dedicated studies are urgently needed.

### Hepatitis E

The hepatitis E virus (HEV) is a small, non-enveloped ssRNA hepevirus that causes over 20 million infections annually. Of its four genotypes, HEV-1 and 2 cause human disease and are spread by feco-oral transmission through contaminated water supply, entering the body via enterocytes and replicating in the liver. This route accounts for the wide geographical variation in disease burden of hepatitis E, which is concentrated in areas of poor sanitation (42, 49). In the general population, hepatitis E is asymptomatic in the vast majority of cases, but still causes 3.3 million symptomatic infections (usually mild, self-limiting and clinically indistinguishable from hepatitis A) and 56,000–70,000 deaths per year, a mortality rate of <0.5% (42).

However, outcomes are much worse for pregnant women: HEV mortality can exceed 50%, particularly if acquired in the third trimester. Several large cohort studies from India have demonstrated that pregnant women with HEV are both more likely to develop fulminant hepatic failure (FHF) and more likely to die from it (237–240). A 2019 systematic review including a total of 1,338 pregnant women with hepatitis E showed a 45% risk of fulminant hepatic failure, a median maternal CFR of 26% and a median fetal CFR of 33%. Other obstetric complications were not consistently reported in the included articles but data from four studies suggest an increased risk of postpartum hemorrhage, ranging from 13.6 to 30% (28). There are no proven drug therapies for hepatitis E (ribavirin, which has shown equivocal benefits in small case series, is contraindicated in pregnancy) and the only available vaccine for prevention of hepatitis E is not manufactured or licensed outside China (28).

Like the other viruses discussed in this review, there is therefore a global imperative to identify the factors that confer increased susceptibility to fatal HEV infection in pregnancy. Common themes emerge: the mechanisms are poorly characterized, difficult to recapitulate through *in vitro* models, influenced by hormonal factors and likely to arise from dysregulation of immune homeostasis at the maternal-fetal interface (241). Studies examining peripheral blood cells and circulating inflammatory mediators from women with HEV-induced FHF have implicated excessive Th2 switching, impaired NFkB-mediated liver regeneration, oxidative stress, and coagulopathy in its pathogenesis; these are reviewed by Perez-Gracia et al. (242), although no convincing individual candidate has emerged. The innate immune response to HEV is also unclear, although it appears to be crucial for prevention of severe disease. In 2015, a comparison of macrophages and DC from pregnant patients with HEV-induced FHF, non-fulminant acute HEV, and healthy pregnant controls found that macrophages from the women who developed fulminant liver disease had significantly impaired phagocytic capacity, with reduced TLR3 and TLR9 expression impeding MyD88-mediated IFN production (239).

Importantly, although the life cycle of HEV remains enigmatic, extrahepatic replication has been demonstrated in both the placenta (241, 243) and, more recently, in cultured human endometrial stromal cells (244). Gouilly et al. (241) showed that HEV-1 causes severe necrotic tissue injury in both decidua and trophoblast, significantly reduces both tissues' ability to produce type III IFNs and distorts the cytokine secretome of cultured DSC, upregulating potent pro-inflammatory mediators including IL-6 and the chemokines CCL-3 and CCL-4. As the authors suggest, these widespread disruptions to the architecture and homeostasis of the maternal-fetal interface are likely to contribute to viral dissemination, adverse obstetric outcomes and increased disease severity in pregnancy.

### Influenza—Seasonal and Pandemic

The *Orthomyxoviridae* family includes three species capable of causing human disease: influenza viruses A, B, and C. Types A and B account for the majority of seasonal influenza, which causes approximately 389,000 deaths per year (245). They have an unusual segmented genome with eight negative-sense RNA strands and, unlike the other viruses discussed in this review, replicate in the nucleus. The pleiomorphic IAV virion consists of a host-derived lipid envelope displaying embedded surface glycoproteins haemagglutinin (HA) and neuraminidase (NA), which account for its antigenicity and are used to classify the different IAV serotypes (e.g., H1N1).

Major influenza pandemics over the last century have consistently shown disproportionately high mortality rates in pregnant women, mostly recently the H1N1 outbreak in 2009 (34–36). A 2011 meta-analysis including a total of 3,110 pregnant women who developed H1N1 showed that rates of hospital admission (52.3%), requirement for intensive care (23.3%) and death (4.1%, of which two thirds occurred in the third trimester) were all significantly increased in pregnancy. Despite only 1% of the susceptible population being pregnant, these women accounted for 5.7% of all deaths in the pooled analysis (52). Although maternal mortality outside a pandemic setting is low, these data prompted the WHO to recommend seasonal influenza vaccination for all pregnant women (52, 246). A subsequent larger systematic review and individual participant data meta-analysis (including 36,498 women of reproductive age) confirmed a 6.8-fold higher risk of requiring hospital admission in pregnant women with H1N1, but did not find any evidence of an increased risk of death (27, 247).

Like Ebola, whether maternal mortality from IAV is actually disproportionately increased remains unclear, but disease is certainly more severe in pregnant women. Pregnant women appear to develop particularly severe lung injury and are also at higher risk of extrapulmonary complications. In a study reporting autopsy findings of 21 Brazilian patients who died of H1N1-related acute respiratory failure, the single pregnant woman in the cohort had the most severe pulmonary disease, with widespread necrotising bronchiolitis, diffuse alveolar damage and significant upregulation of TLR3, IFN-γ, and granzyme B-producing cells in the airway epithelium, suggesting excessive activation of the innate immune response (248).

Mouse models have shown that disrupted TLR signaling is an important component of IAV pathogenesis. Following cell entry, IAV virions are internalized into endosomes, in which the low pH induces a conformational shift in HA resulting in release of the nucleocapsid protein into the cytosol. This process is essential for viral replication but also stimulates the innate immune response: in pDC isolated from mouse spleens, endosomal sensing of IAV ssRNA by TLR7 led to massive IFN-α release, a response that was completely abrogated in TLR7^−/−^ mice (129). TLR3-deficient mice also show a muted response to IAV infection, with significant reductions in pro-inflammatory cytokine expression and CD8^+^ T cell recruitment to the broncho-alveolar space. However, despite higher viremic burden, this actually confers a survival advantage compared to control TLR3-competent mice (249). In purified human alveolar epithelial cells, RIG-I and TLR3 are the primary IAV sensors and trigger a type III-predominant IFN response (250). As with the other RNA viruses discussed here, IAV has a sophisticated array of innate evasion mechanisms, which are reviewed by Kikkert (251). IAV can also induce necroptosis of infected cells, leading to potent neutrophil recruitment and exacerbated lung injury. Mice deficient in MLKL, a critical mediator of necroptosis, exhibit improved survival in response to a lethal IAV challenge (252).

Pregnancy appears to suppress the systemic type I and type III IFN responses to IAV. A 2012 study showed that peripheral blood mononuclear cells from pregnant women exhibited a 10-fold reduction in the expression of protein kinase receptor, an ISG that is stimulated early in the antiviral response, compared to matched female controls in response to H1N1 IAV challenge. This effect was partially reversed by vaccination, although still not completely restored to non-pregnant levels (206). Some studies have also shown an IAV-induced upregulation of T cell costimulatory markers on pDC, hypothesizing that the virus may break the physiological attenuation of DC maturation in pregnancy and lead to exaggerated immune responses and tissue damage; conversely, others suggest that a failure to activate lung DC and recruit virus-specific CD8 T cells to the airway epithelium could contribute to the inability to control the virus in pregnancy (253, 254). Whether the severe disease observed in pregnancy results from a failure to generate robust innate responses or a harmful virus-induced disruption of the tolerogenic state remains to be clarified.

The role of hormones in modulating lung physiology and the response to IAV has been extensively studied. Progesterone, which is essential for the maintenance of pregnancy, is a muscle relaxant that causes physiological airway dilatation. It also affects the innate immune system, preventing NK cell degranulation and upregulating neutrophil-attractant chemokines in the respiratory epithelium. It interacts closely with the prostaglandins PGE2, which increases vascular permeability in the lungs and downregulates effector mechanisms in neutrophils and macrophages, and PGF2a, a potent broncho- and vasoconstrictor (86). In animal studies, female mice treated with exogenous progesterone are protected against severe IAV disease through increased expression of amphiregulin, which promotes tissue repair in the lungs and improves survival (255). However, the immunoregulatory role of progesterone appears to be strain- and exposure-specific: in another study, pre-treatment of female mice with progesterone protected them against severe disease during initial H1N1 exposure but reduced their survival during subsequent challenge with H3N2 (256). Influenza infection in pregnant mice dysregulates the progesterone-prostaglandin axis, leading to bronchoconstriction, an alveolar influx of neutrophils and respiratory failure (86). The implications of these findings in the severity of influenza in pregnancy are discussed further in a recent review in this journal by Littauer and Skountzou (38).

### Novel Coronaviruses

Coronaviruses, which were first isolated from humans in the 1960s, cause frequent mild upper respiratory tract illnesses on a large scale but are not usually associated with significant disease. However, in the last 20 years, three zoonotic novel coronaviruses capable of causing severe pneumonia have emerged: severe acute respiratory syndrome coronavirus (SARS-CoV), Middle East respiratory syndrome coronavirus (MERS-CoV) and COVID-19 (SARS-CoV-2). Global experiences of the first two, which disproportionately affected pregnant women, have caused significant apprehension about the consequences of the third, which remains subject to intensive and ongoing research (55, 257). Given that pneumonia is the leading non-obstetric infectious cause of maternal death, vigilance is required with any emerging respiratory virus and pregnant women should be prioritized in efforts to anticipate and mitigate their effects (258).

SARS-CoV caused over 8,400 cases in 29 countries in the outbreak of 2002–2003. The 916 reported deaths corresponded to a global CFR of ~11%. Studies describing its effects in pregnant women, although small, are strongly suggestive of a higher burden of mortality and morbidity than in the general population. Wong et al. summarized outcomes in 12 pregnant women hospitalized due to confirmed SARS-CoV in Hong Kong in 2003: 50% were admitted to intensive care due to hypoxia (vs. 20% of non-pregnant patients), 33% required mechanical ventilation and 25% died. Associated complications included disseminated intravascular coagulation, renal failure and superimposed bacterial pneumonia. Of the 7 who presented in the first trimester, 5 had a spontaneous miscarriage; and of the 5 who presented beyond 24 weeks' gestation, 80% delivered preterm. Surviving neonates did not develop SARS and there was no evidence of vertical transmission, although placental histopathology did show other features indicative of maternal hypoxemia. The authors note that the use of ribavirin in 11/12 cases (following careful counseling about potential teratogenicity) may have exacerbated the high observed rate of first-trimester miscarriage (57). A case-control study, also from Hong Kong, confirmed the increased severity of disease in pregnancy by comparing 10 pregnant to 40 non-pregnant women with SARS at the same hospital: death (30 vs. 0%), intensive care admission (60 vs. 17.5%) and renal failure were all significantly more common in the pregnant group (58).

MERS-CoV, which emerged in 2012, caused 2494 confirmed cases and 858 deaths, with a CFR of 34.4%. The higher mortality rate has been attributed to both poor infection control, facilitating rapid propagation in healthcare settings, and an inherently more aggressive clinical course, with faster progression to respiratory failure. The clinical spectrum was broader than in SARS, with immunocompetent adults often reporting mild or moderate symptoms while deaths were more concentrated in those who were immunosuppressed, pregnant or had major medical comorbidities (55, 259). A 2019 synthesis of 11 pregnant women with MERS-CoV found that 54% required intensive care and 27% died. Although this was not elevated compared to the CFR in the general population, these numbers are too small to provide confident assurance that pregnant women are not at increased risk from MERS-CoV. As with SARS, no episodes of vertical transmission were documented, although umbilical cord and neonatal blood sampling were not universally performed (59).

The spread of COVID-19 (SARS-CoV-2) has vastly exceeded that of SARS and MERS, with over 21 million cases and 760,000 deaths at the time of writing (260). However, its mortality rate is considerably lower than the two previous novel coronavirus outbreaks, as is the proportion of patients who develop severe disease (pooled estimate 18.0%) (261).

In the early stages of its escalation, experience from the SARS-CoV and MERS-CoV outbreaks prompted some national health policy departments to recommend increased caution in pregnant women. However, accumulating data are reassuring on this front: a systematic review of 2,567 affected pregnancies published in July showed a 7% intensive care unit admission rate with maternal mortality of approximately 1% (262) and a population-level analysis of 427 pregnant women hospitalized with COVID-19 in the UK showed no evidence of an increased risk of severe disease compared to the general hospital population (60). In contrast to SARS-CoV and MERS-CoV, vertical transmission appears to occur in a small proportion of cases of SARS-CoV-2: in a recent meta-analysis, 3.2% of neonates tested positive on nasopharyngeal swabs and 3.7% had positive serology, based on IgM positivity (263), however, caution is required given that the validity of IgM serology tests can be compromised by cross-reactivity. These findings are supported by the recent systematic confirmation of transplacental transmission of SARS-CoV-2 following maternal infection at 35 weeks' gestation (264).

The exponential transmission of COVID-19 has prompted renewed efforts to identify integral components of the immune response to coronaviruses. While innate host defenses rely on the same cardinal mechanisms as for the other RNA viruses discussed—namely, the interferon response—coronaviruses appear to have evolved a particularly diverse repertoire of innate immune evasion strategies. Inhaled coronaviruses enter pneumocytes and macrophages in the upper respiratory tract and replicate in the cytosol, hijacking host intracellular membranes to create “replication organelles” (RO) that facilitate viral replication while simultaneously shielding the RNA within double-membrane vesicles to prevent detection by intracellular PRRs such as RIG-I (265).

Coronaviruses go to great lengths to avoid recognition: their non-structural proteins even exhibit endonuclease activity, allowing them to degrade their own RNA and forestall activation of PAMP-mediated antiviral signaling (266). As well as these evasion strategies, coronaviruses can also directly antagonize the innate immune response: the SARS-CoV non-structural protein-1 (NSP-1) binds to the 40S subunit of host ribosomes and induces “translational shutoff,” bringing all cellular expression of antiviral effector proteins to a halt (267). This particularly intricate capacity of CoV to both elude and suppress innate effector mechanisms may contribute to their pandemic potential. However, no studies thus far have been specifically designed to investigate the disparities between individual coronaviruses in terms of their effects on maternal disease and vertical transmission in pregnancy.

## Challenges and Future Developments

Determining exactly how innate immune mechanisms and corresponding viral evasion strategies contribute to the disproportionately severe disease observed in pregnancy is an ongoing problem. Challenges range from the technical and ethical (e.g., the difficulty of accessing human decidual tissue and replicating human pregnancy in animal models) to the global, with the ever-present threat of widespread outbreaks caused by novel viruses with pandemic potential. Emerging viruses, epitomized by the current COVID-19 pandemic and the ongoing Ebola outbreak in North Kivu, highlight the vulnerability and inequity in the global health infrastructure. However, there is also a considerable threat from resurgence of previously controlled viruses: the global re-emergence of measles since 2016, including in several countries where transmission had previously been eradicated, has caused thousands of deaths and poses a major risk to pregnant women (268). A 2017 UK case report described a patient who required emergency Cesarean section and extracorporeal membrane oxygenation for deteriorating respiratory failure at 32 weeks' gestation (269). Control of preventable viral diseases like measles depends on high rates of vaccination coverage, which is compromised by conflict, migration, and persistent belief in the discredited association with autism (268).

The ability to protect vulnerable groups from viral infection depends partly on public health measures such as vaccination and vector control but also on our understanding of the biological correlates of this vulnerability. The advent of technologies such as mass cytometry and single-cell RNA sequencing have already shown promise (270), offering unparalleled insights into the cellular architecture of the maternal-fetal interface in early pregnancy (183, 271, 272). These, along with the recent development of human trophoblast organoids that replicate the placental secretome *in vitro*, may transform our ability to study the effects of viral infection in pregnancy (273).

## Conclusion

**Figure 5:**
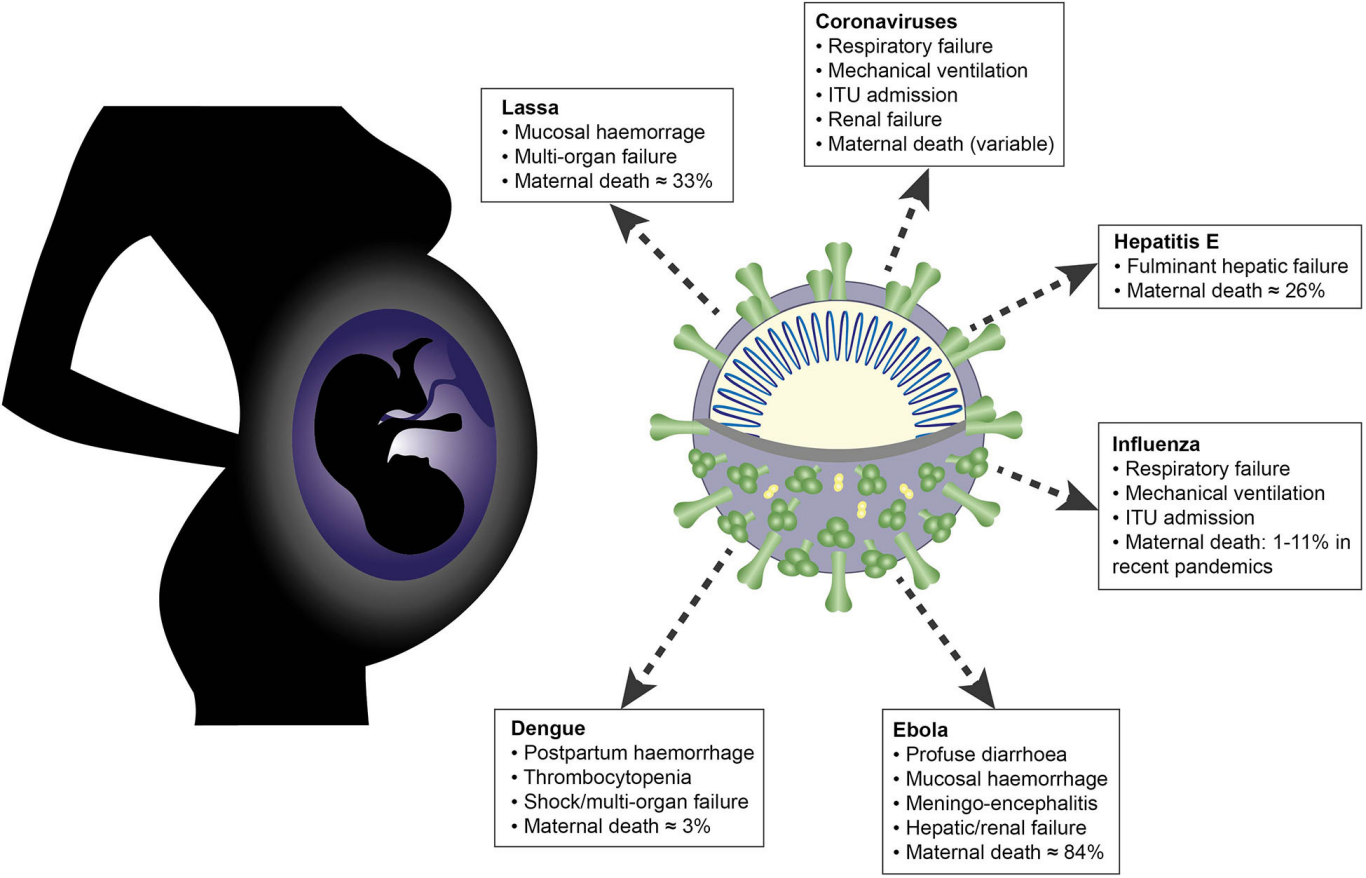
Schematic summarizing effects of maternal infection with the following RNA viruses during pregnancy: Lassa, Ebola, dengue, hepatitis E, influenza and novel coronaviruses.

The RNA viruses discussed in this article share many overlapping features in their clinical manifestations and their interactions with the maternal immune system (Figure 5). Common themes include placental tropism, an association with adverse obstetric outcomes and the importance of a tightly regulated interferon response, which is reflected in the evolution of diverse viral IFN evasion mechanisms. Innate immune cells are uniquely positioned at the maternal-fetal interface and orchestrate the balance between the conflicting immunological priorities of pregnancy: tolerance of the fetal allograft and defense of both mother and fetus against invasive pathogens.

The disproportionate rates of severe disease and mortality observed in pregnant women with Lassa, Ebola, dengue, hepatitis E, influenza and certain novel coronavirus infections cannot be attributed purely to immunological adaptations: social and behavioral factors also contribute (212). However, it is only through research that is tailored toward these pregnancy-specific factors that the outcomes will improve. In particular, as recently and eloquently argued by Gomes et al. (274) and Schwartz and Graham (55), the systematic exclusion of pregnant women from the design of vaccine trials for viral illnesses will perpetuate and exacerbate the problem. Addressing the gaps in our knowledge of innate immunity in pregnancy is an urgent priority.

## Author Contributions

EC and TM conceived the article. EC wrote the first draft of the manuscript. TM wrote sections of the manuscript, provided critical revisions, and edited the text. FÅ generated all the figures for the manuscript. DW and IF provided critical revisions of the manuscript and edited the text. All authors contributed to manuscript revision, read, and approved the submitted version.

## Conflict of Interest

The authors declare that the research was conducted in the absence of any commercial or financial relationships that could be construed as a potential conflict of interest.
